# The interaction of thrombocytopenia, hemorrhage, and platelet transfusion in venoarterial extracorporeal membrane oxygenation: a multicenter observational study

**DOI:** 10.1186/s13054-023-04612-5

**Published:** 2023-08-21

**Authors:** Senta Jorinde Raasveld, Claudia van den Oord, Jimmy Schenk, Walter M. van den Bergh, Annemieke Oude Lansink - Hartgring, Franciska van der Velde, Jacinta J. Maas, Pablo van de Berg, Roberto Lorusso, Thijs S. R. Delnoij, Dinis Dos Reis Miranda, Erik Scholten, Fabio Silvio Taccone, Dieter F. Dauwe, Erwin De Troy, Greet Hermans, Federico Pappalardo, Evgeny Fominskiy, Višnja Ivancan, Robert Bojčić, Jesse de Metz, Bas van den Bogaard, Dirk W. Donker, Christiaan L. Meuwese, Martin De Bakker, Benjamin Reddi, José P. S. Henriques, Lars Mikael Broman, Dave A. Dongelmans, Alexander P. J. Vlaar

**Affiliations:** 1 Department of Critical Care, Amsterdam University Medical Centers, Location Academic Medical Centers, Meibergdreef 9, 1105 AZ Amsterdam, The Netherlands; 2 Department of Cardiology, Amsterdam University Medical Centers, Location Academic Medical Centers, Amsterdam, The Netherlands; 3 Department of Critical Care, University Medical Center Groningen, University of Groningen, Groningen, The Netherlands; 4 Adult Intensive Care Unit, Leiden University Medical Center, Leiden, The Netherlands; 5 Adult Intensive Care Unit, Catharina Hospital Eindhoven, Eindhoven, The Netherlands; 6 Cardiothoracic Surgery Department, Heart and Vascular Center, Maastricht University Medical Center, and Cardiovascular Research Institute Maastricht (CARIM), Maastricht, The Netherlands; 7 Department of Cardiology, Maastricht University Medical Center, Maastricht, The Netherlands; 8 Adult Intensive Care Unit, Erasmus University Medical Center, Rotterdam, The Netherlands; 9 Department of Intensive Care, St. Antonius Hospital, Nieuwegein, The Netherlands; 10 Department of Intensive Care, Université Libre de Bruxelles, Hôpital Erasme Bruxelles, Brussels, Belgium; 11 Surgical Intensive Care Unit, Department of Intensive Care Medicine, University Hospital Leuven, Leuven, Belgium; 12 Medical Intensive Care Unit, Department of General Internal Medicine, University Hospitals Leuven, Herestraat 49, 3000 Leuven, Belgium; 13 Laboratory of Intensive Care Medicine, Department of Cellular and Molecular Medicine, KU Leuven, Herestraat 49, 3000 Leuven, Belgium; 14 Department of CardioThoracic and Vascular Anesthesia and Intensive Care, AO SS Antonio e Biagio e Cesare Arrigo, Alessandria, Italy; 15 Department of Anesthesia and Intensive Care, IRCCS San Raffaele Scientific Institute, Milan, Italy; 16 Department of Anesthesia and Intensive Care, University Hospital Centre Zagreb, Zagreb, Croatia; 17 Department of Intensive Care, OLVG, Amsterdam, The Netherlands; 18 Intensive Care Center, University Medical Center Utrecht (UMCU), Utrecht, The Netherlands; 19 Department of Critical Care, Royal Adelaide Hospital, Adelaide, Australia; 20 ECMO Center Karolinska, Karolinska University Hospital, Stockholm, Sweden; 21 Cardiovascular and Respiratory Physiology, TechMed Centre, University of Twente, Enschede, The Netherlands; 22 Department of Intensive Care, Maastricht University Medical Center, Maastricht, The Netherlands; 23 Department of Epidemiology and Data Science, Amsterdam University Medical Centre, Amsterdam Public Health, University of Amsterdam, Location AMC, Amsterdam, The Netherlands

**Keywords:** Thrombocytopenia, Platelet transfusion, Hemorrhage, Venoarterial extracorporeal membrane oxygenation

## Abstract

**Background:**

Thrombocytopenia, hemorrhage and platelet transfusion are common in patients supported with venoarterial extracorporeal membrane oxygenation (VA ECMO). However, current literature is limited to small single-center experiences with high degrees of heterogeneity. Therefore, we aimed to ascertain in a multicenter study the course and occurrence rate of thrombocytopenia, and to assess the association between thrombocytopenia, hemorrhage and platelet transfusion during VA ECMO.

**Methods:**

This was a sub-study of a multicenter (*N* = 16) study on transfusion practices in patients on VA ECMO, in which a retrospective cohort (Jan-2018–Jul-2019) focusing on platelets was selected. The primary outcome was thrombocytopenia during VA ECMO, defined as mild (100–150·10^9^/L), moderate (50–100·10^9^/L) and severe (< 50·10^9^/L). Secondary outcomes included the occurrence rate of platelet transfusion, and the association between thrombocytopenia, hemorrhage and platelet transfusion, assessed through mixed-effect models.

**Results:**

Of the 419 patients included, median platelet count at admission was 179·10^9^/L. During VA ECMO, almost all (*N* = 398, 95%) patients developed a thrombocytopenia, of which a significant part severe (*N* = 179, 45%). One or more platelet transfusions were administered in 226 patients (54%), whereas 207 patients (49%) suffered a hemorrhagic event during VA ECMO. In non-bleeding patients, still one in three patients received a platelet transfusion. The strongest association to receive a platelet transfusion was found in the presence of severe thrombocytopenia (adjusted OR 31.8, 95% CI 17.9–56.5). After including an interaction term of hemorrhage and thrombocytopenia, this even increased up to an OR of 110 (95% CI 34–360).

**Conclusions:**

Thrombocytopenia has a higher occurrence than is currently recognized. Severe thrombocytopenia is strongly associated with platelet transfusion. Future studies should focus on the etiology of severe thrombocytopenia during ECMO, as well as identifying indications and platelet thresholds for transfusion in the absence of bleeding.

*Trial registration*: This study was registered at the Netherlands Trial Registry at February 26th, 2020 with number NL8413 and can currently be found at https://trialsearch.who.int/Trial2.aspx?TrialID=NL8413.

**Supplementary Information:**

The online version contains supplementary material available at 10.1186/s13054-023-04612-5.

## Background

Hemorrhage is an important and frequent complication during extracorporeal membrane oxygenation (ECMO). Although changes in anticoagulation strategies and improved circuit technology have led to a reduction in its occurrence rate, it remains a significant problem attributing to worse patient outcomes [1]. Active bleeding is one of the main indications to supply coagulation factors or transfuse blood products, including platelets. Guidelines by the Extracorporeal Life Support Organization (ELSO) state that as a spontaneous bleeding can occur at a platelet count of 20·10^9^/L, the advised threshold for platelet transfusion is 80·10^9^/L [2]. However, evidence-based guidelines are lacking, resulting in a wide range of thresholds used in daily practice [3].

Thrombocytopenia, defined as a platelet count < 150·10^9^/L [4], is common in patients supported with venoarterial ECMO (VA ECMO). Approximately one in five patients develops a thrombocytopenia during their ECMO support, independent of ECMO duration [5, 6]. Its etiology is complex and multifactorial, consisting of platelet activation and consumption by the extracorporeal circuit, hemodilution, hemorrhage, and the usage of platelet count and function altering medication such as platelet inhibitors and heparin [2, 7]. Importantly, thrombocytopenia is found to be a risk factor for hemorrhage and even mortality [8, 9].

In VA ECMO, the platelet transfusion rate has been described in a few retrospective studies, showing a range of 4% up to 62% [8, 10, 11], and has been associated with an increased risk of mortality [8]. As such, hemorrhage, thrombocytopenia and platelet transfusion are intertwined and associated with mortality. However, up to now, most studies are limited to a single-center design, therefore limiting generalizability. Moreover, correction for confounding factors is often limited or insufficiently described. Although in 2020 a meta-analysis was performed, conclusions were limited due to the high level of heterogeneity, as well as the very limited number of studies describing thrombocytopenia or platelet transfusion in VA ECMO [5].

Therefore, the aim of this international multicenter study was to ascertain the course and occurrence rate of thrombocytopenia, and to assess the association between thrombocytopenia, hemorrhage and platelet transfusion during VA ECMO.

## Methods

This was a scheduled sub-study of a multicenter (*N* = 16) mixed-method study on transfusion practices in patients receiving ECMO in intensive care units (ICUs) worldwide, combining a retrospective cohort with a survey focusing on local transfusion practices. All patients, aged 18 years and older, who received ECMO between January 1, 2018, and July 1, 2019, were included. This sub-study specifically focuses on platelet transfusion in VA ECMO, therefore, all other modes as well as patients with an ECMO run of less than 24 h were excluded. The study did not comply with the requirements as stated in the Medical Research Involving Human Subjects Act (WMO), as such, it received a waiver by the institutional review board (IRB) of the Amsterdam University Medical Centers, location Academic Medical Centers (AMC: W19_222#19.267), and thereafter, if required, as well by local IRBs. This study was registered on the Netherlands Trial Register (NL8413, date of registration 26-Feb-2020).

### Retrospective cohort: data collection

Data were collected retrospectively using electronic patient records, consisting of patient demographics, ECMO characteristics, laboratory values, transfusion parameters, and patient outcomes. ECMO characteristics included the primary indication for VA ECMO, cannulation site (central versus peripheral), type of insertion (percutaneously versus surgically), and in case of peripheral cannulation, if a distal cannula was placed as well. Indications for ECMO were further divided into post-cardiotomy, acute myocardial infarction and other. Laboratory values and transfusion parameters were collected on a daily basis during ECMO until decannulation or a maximum of 28 days, whatever came first. Laboratory values consisted of lowest hemoglobin levels (Hb), lowest platelet count, highest aPTT and lowest fibrinogen level measured on that calendar day. Transfusion parameters consisted of whether a transfusion occurred and if so, how many units were transfused. Lastly, patient outcomes consisted of complications (i.e., hemorrhage, thrombotic events, acute kidney injury), and 28-day mortality. When available, the definitions used were in line with definitions used in the ELSO registry. Exceptions are stated in the list of definitions provided in the Additional file 1.

### Survey: institutional transfusion and anticoagulation management

In addition to the retrospective data collection, a survey was created by MK and JR assessing transfusion and anticoagulation practices in the participating centers. This questionnaire, included in the Additional file 1, contained information on the thresholds for blood (product) transfusion and type of product used.

### Outcome measures

The primary outcome was the occurrence of thrombocytopenia during ECMO, defined as a nadir platelet count < 150·10^9^/L. Thrombocytopenia was divided into three degrees of severity: mild (100–150·10^9^/L), moderate (50–100·10^9^/L) and severe (< 50·10^9^/L), in line with previous research [12]. Other outcomes included the time course of thrombocytopenia, the occurrence rate of platelet transfusion, number of transfusions received, concomitant RBC transfusion, and the association between thrombocytopenia, hemorrhage and platelet transfusion.

### Statistical analyses

R (version 4.2.2) within the Rstudio interface was used for the statistical analyses. Descriptive statistics were reported as mean and standard deviation (± SD) or median [1st–3rd quartile] when appropriate. To compare the different subgroups of thrombocytopenia severity, as well as for the subgroup analyses comparing either bleeding versus non-bleeding or transfused versus non-transfused patients, either a Mann–Whitney *U* test or Chi-square test was used. A post hoc test with *P*-value adjustment according to Benjamini–Hochberg was applied when considered appropriate. For all analyses, a two-sided *P*-value < 0.05 was considered statistically significant.

To assess the association between thrombocytopenia, hemorrhage and platelet transfusion, mixed-effects models were used. Platelet transfusion was considered the chronological effect of either hemorrhage or thrombocytopenia, and thus used as the dependent outcome. To define the unadjusted effect, a reduced model was applied to assess the effect of either hemorrhage or severity of thrombocytopenia on receiving a platelet transfusion, solely correcting for ECMO duration and center. To further adjust for confounding, an advanced model was developed using center and duration as random effect, as well as an a priori defined set of confounders as fixed effects. These confounders were identified in the literature and included: sex, age, history of cardiovascular disease, SOFA score at day of ECMO initiation, cannulation site (reference: peripheral), daily aPTT, a thrombotic complication during ECMO and anticoagulation type (reference: unfractionated heparin). In addition to this advanced model, a final model was created including an interaction term combining hemorrhage and thrombocytopenia. This interaction term included thrombocytopenia with hemorrhage, thrombocytopenia without hemorrhage, and hemorrhage without thrombocytopenia. Odds ratios (OR) were presented with their 95% confidence interval (95% CI).

### Handling missing data

Missing data were assessed after data collection. Patterns in missing data were analyzed (i.e., missing-at-random, not-missing-at-random, missing-completely-at-random) and variables containing more than 50% missing values were excluded from the dataset, which did not result in the exclusion of any of the pre-defined covariates of interest. Missing data were not imputed, since the employed mixed-effect models use maximum-likelihood estimation to handle missing data.

## Results

Of the total of 433 patients, 419 were eligible for further analyses (Additional file 1: S3. Flowchart). At ICU admission, median platelet count was 179·10^9^/L [119–253·10^9^/L]. An overview of baseline characteristics can be found in Table 1. To highlight, most patients had a peripheral cannulation configuration (*N* = 356, 86%), and over half of the patients was cannulated using a surgical technique (*N* = 227, 56%). The nadir platelet count at the first day of ECMO showed an average 49·10^9^/L [6–102·10^9^/L] decrease when compared to the last known value before cannulation.

**Table 1:** Baseline demographics, stratified by nadir platelet count during ECMO

Variable	Overall (*N* = 419)	Normal (*N* = 20)	Mild (*N* = 60)	Moderate (*N* = 159)	Severe (*N* = 179)	*P*-value	Post hoc
Age, years	57 [47–66]	55 [46–61]	57 [43–67]	58 [47–67]	57 [47–67]	0.43	n.s
BMI, kg/m^2^	27.0 [24.3–30.6]	29.5 [26.2–32.0]	26.9 [24.0–30.7]	27.1 [24.6–30.4]	26.8 [24.2–30.4]	0.22	n.s
Female	154 (37)	7 (35)	19 (32)	48 (30)	80 (45)	0.04	Severe versus moderate*
*Medical history*							
Hypertension	139 (62)	7 (35)	13 (25)	53 (33)	66 (37)	0.74	n.s
Diabetes mellitus	68 (30)	8 (40)	13 (25)	20 (13)	27 (15)	< 0.001	Severe versus mild*/normal** moderate versus normal**
Myocardial infarction	76 (34)	4 (20)	9 (15)	32 (20)	31 (17)	0.76	n.s
Asthma/COPD	37 (55)	3 (15)	6 (10)	19 (12)	9 (5)	0.22	n.s
Chronic kidney disease	25 (6)	0 (0)	4 (7)	7 (4)	14 (8)	0.38	n.s
*Day of ECMO initiation*							
SOFA-score	11 [8–13]	9 [8–10]	10 [8–12]	10 [8–13]	13 [10–14]	< 0.001	Severe versus mild/moderate/normal**
Lactate, mmol/L	5.2 [2.4–10.0]	3.6 [1.6–5.6]	2.5 [1.8–8.1]	4.2 [2.1–12]	6.6 [3.4–10.4]	< 0.01	Severe versus mild/normal*
Hb, g/dL	11.6 [9.5–13.5]	11.4 [9.5–13.7]	12.0 [10.3–13.7]	11.8 [9.3–13.6]	11.0 [9.4–13.4]	0.59	n.s
Platelet count	179 [119–253]	324 [254–379]	216 [155–264]	199 [142–255]	133 [82–197]	< 0.001	Moderate versus normal *** mild versus normal *** severe versus mild/moderate/normal***
Difference in platelet count day of initiation -nadir platelet count at first day of ECMO	49 [6–102]	66 [− 1 to 105]	28 [0–66]	50 [9.5–93]	51 [16–112]	0.42	n.s
*ECMO characteristics*							
Duration, days	5 [3–8]	3 [2–4]	4 [2–7]	4 [3–8]	6 [4–9]	< 0.001	Moderate versus normal* severe versus mild**/moderate*/normal**
Second run	38 (9)	1 (5)	3 (5)	12 (8)	22 (12)	0.23	n.s
Peripheral cannulation configuration	355 (86)	20 (100)	55 (92)	133 (84)	147 (84)	0.10	n.s
Surgical cannulation	226 (56)	11 (55)	23 (40)	86 (56)	106 (60)	0.06	n.s
Distal leg perfusion cannula	288 (71)	15 (79)	42 (72)	106 (69)	125 (71)	0.84	n.s
ECPR	106 (26)	3 (15)	20 (33)	41 (26)	42 (24)	0.36	n.s
Main reason of ECMO initiation						0.04	n.s
Acute myocardial infarction	116 (28)	5 (25)	15 (25)	49 (31)	47 (26)		
Post-cardiotomy	113 (27)	1 (5)	11 (18)	43 (27)	58 (32)		
Other	189 (45)	14 (70)	34 (57)	67 (42)	74 (41)		

BMI, body mass index; COPD, chronic obstructive pulmonary disease; ECMO, extracorporeal membrane oxygenation; ECPR, extracorporeal cardiopulmonary resuscitation; Hb, hemoglobin; SOFA, sequential organ failure assessment

*P < 0.05, **P < 0.01, ***P < 0.001, n.s., non-significant (*P* > 0.05)

**Fig. 1:**
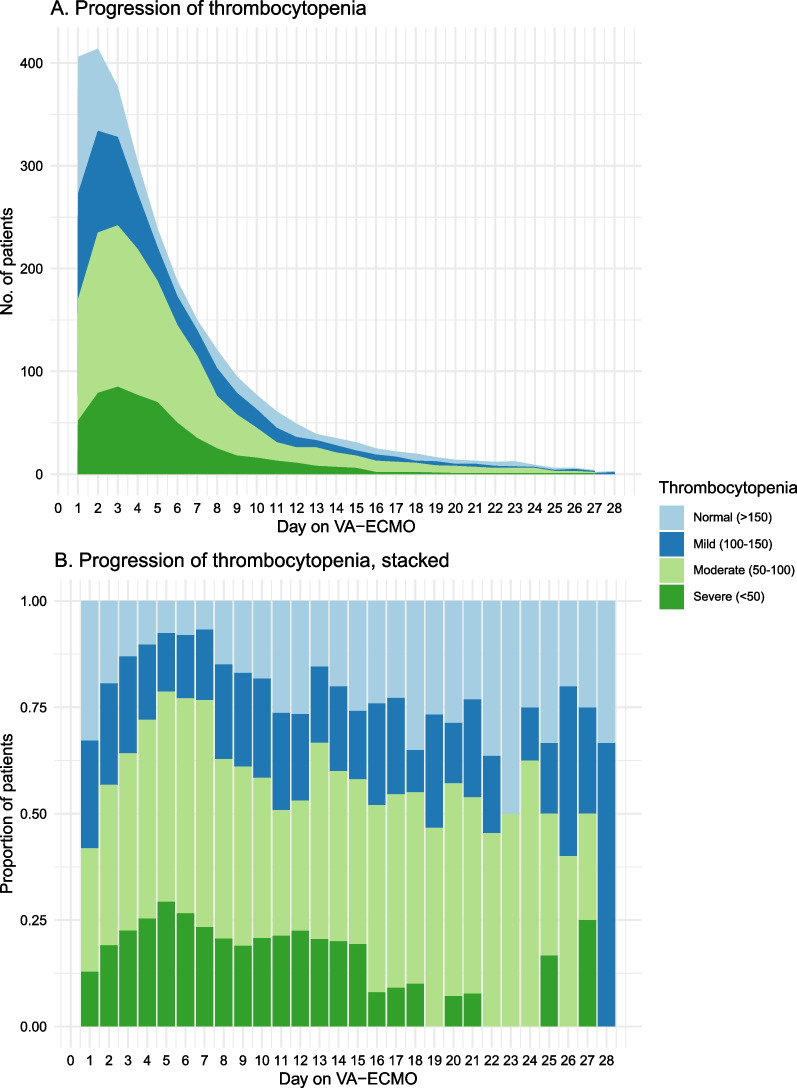
Progression of thrombocytopenia over time. This figure describes the proportion of patients suffering from a thrombocytopenia during VA ECMO. In Panel (**A**), the y-axis shows the absolute number of patients. In Panel (**B**), a stacked bar-plot shows the proportion of patients per day with a certain degree of thrombocytopenia. No., number, VA ECMO, venoarterial extracorporeal membrane oxygenation

**Fig. 2:**
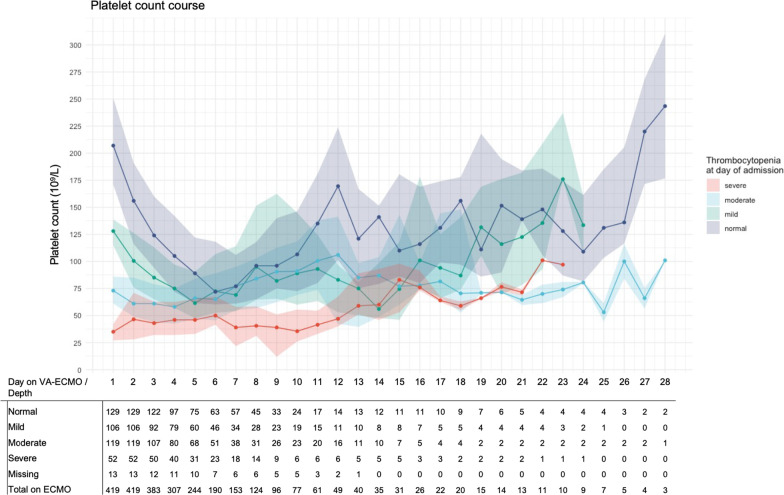
Course of platelet count over time, stratified by severity of thrombocytopenia at admission. This figure describes the course of platelet count over time as median with 1st–3rd quartile, as stratified by the degree of severity of thrombocytopenia as measured at the day of admission (before VA ECMO). VA ECMO, venoarterial extracorporeal membrane oxygenation

Nearly all patients developed a thrombocytopenia during ECMO (398/418, 95%), of which two third already during the first day (273/406, 67%, missing = 13). During ECMO, lowest platelet count was < 50·10^9^/L in 179 patients (43%), 50–100·10^9^/L in 159 patients (38%), and 100–150·10^9^/L in 60 patients (14%). An overview of the degree of thrombocytopenia is shown in Fig. 1. The largest proportion of severely thrombocytopenic patients was found at day 5, when 70 out of the remaining 244 patients had a platelet count < 50·10^9^/L (29%). With the exception of the yet severely thrombocytopenia patients, the platelet course showed an initial decrease in platelet count, followed by a stabilization and further platelet recovery, as shown in Fig. 2. This same trend was found independent of overall hemorrhage and transfusion status (Additional file 1: Fig. S1).

In comparison with the patients with a nadir platelet count > 100·10^9^/L, patients that developed a severe thrombocytopenia during ECMO had a higher lactate level, higher SOFA score and lower platelet count before ECMO initiation (all adjusted *P* < 0.05). In addition, with a median of 6 days [4–9], the total days of ECMO were longest in patients with a severe thrombocytopenia during ECMO (adjusted *P* < 0.01). Unadjusted 28-day mortality was highest in patients that suffered a severe thrombocytopenia, significantly lower than patients with other degrees of thrombocytopenia (*P* < 0.01).

### Institutional anticoagulation and transfusion management

Almost all centers used unfractionated heparin as standard anticoagulation, with the exception of one center that used bivalirudin in all patients. Platelet transfusion thresholds varied from 10 to 100·10^9^/L, whereas half of the centers listed a threshold of 50·10^9^/L. One third of the centers stated different thresholds for bleeding versus non-bleeding patients, resulting in an increased threshold for platelet transfusion in the presence of bleeding. The proportion of patients that received a platelet transfusion ranged from 25 to 92% among the centers (Additional file 1: Table S1). No association was found between the threshold and transfusion rate among the centers (*P* = 0.49).

### Platelet transfusion and hemorrhage

Overall, 226 patients (54%) received one or more platelet transfusions during ECMO, at a median of 2 days [1–3] of the total ECMO duration of 5 days [3–8]. Per day with a transfusion, one unit of platelets [1–2] was administered, adding up to a total of 4 units [2–7]. Of the patients that developed a severe thrombocytopenia, 4 out of 5 received a platelet transfusion during ECMO (*N* = 146/179, 82%). When the nadir platelet count during ECMO decreased, the number of days with a transfusion as well as the total amount transfused increased (Table 2). In comparison with patients that did not receive a transfusion, patients that were transfused were more likely to be female, had a longer ECMO run, and had a lower platelet count before as well as during ECMO (Additional file 1: Table S2, S3). Lastly, the proportion that received either a transfusion of red blood cells and plasma, or was given fibrinogen or tranexamic acid, increased with the depth of thrombocytopenia (Additional file 1: Table S4.).

**Table 2:** Platelet course, transfusion and complications

Variable	Overall (*N* = 419)	Normal (> 150) (*N* = 20)	Mild (100–150) (*N* = 60)	Moderate (50–100) (*N* = 159)	Severe (< 50) (*N* = 179)	*P*-value	Post hoc
*Laboratory values*							
Platelet count prior	179 [119–253]	324 [254–379]	216 [155–264]	199 [142–255]	133 [82–197]	< 0.001	Severe versus mild/moderate/normal*** normal versus mild/moderate**
Difference platelet count before-after cannulation	49 [6–102]	66 [− 1 to 105]	28 [0–66]	50 [10–93]	51 [16–112]	0.42	n.s
During ECMO: minimal platelet count	56 [37–89]	204 [167–240]	118 [109–128]	68 [58–81]	34 [23–42]	< 0.001	Severe versus mild/moderate/normal*** normal versus mild/moderate**
During ECMO: mean platelet count	89 [63–129]	228 [198–292]	146 [1357–169]	100 [84–116]	61 [47–75]	< 0.001	Severe versus mild/moderate/normal*** normal versus mild/moderate**
Day of nadir platelet count	4 [1–5]	3 [2–4]	3 [2–5]	4 [2–5]	4 [2–5]	0.93	n.s
*Platelet transfusion*							
Proportion transfused	226 (54)	0 (0)	8 (13)	72 (45)	146 (82)	< 0.001	Severe versus mild/moderate/normal*** normal versus moderate**
Total platelets transfused	4 [2–7]	–	1 [1–1.25]	3 [1–5]	4.50 [2–10]	< 0.001	Severe versus mild/moderate/normal*** normal versus moderate**
No. of days receiving platelet transfusion	2 [1–3]	–	1 [1–1.25]	1 [1–2]	2 [1–4]	< 0.001	Severe versus mild/moderate/normal*** normal versus moderate**
Number of platelets transfused per day on ECMO (units)	0.63 [0.33–1.14]	–	0.50 [0.31–0.50]	0.41 [0.25–0.86]	0.75 [0.44–1.31]	< 0.01	Severe versus mild/moderate/normal*** normal versus moderate**
*Number of platelets transfused per transfusion day (units)*^a^							
1	306/579 (53)	–	8/10 (80)	70/124 (56)	228/445 (51)	0.13	n.s
2–3	179/579 (31)	–	2/10 (20)	30/124 (24)	147/445 (33)	0.13	n.s
≥ 4	94/579 (16)	–	0 (0)	24/124 (19)	70/445 (16)	0.27	n.s
Concomitant RBC transfusion among the transfused	221 (98)	–	8 (100)	69 (96)	144 (99)	0.38	n.s
*Complications*							
Acute kidney injury	242 (58)	8 (40)	28 (47)	89 (56)	117 (65)	0.02	n.s
Hemorrhage	207 (49)	1 (5)	14 (23)	77 (48)	115 (64)	< 0.001	Severe versus mild/moderate/normal*** moderate versus mild**/normal***
Thrombotic event	112 (27)	5 (25)	9 (15)	32 (20)	66 (37)	0.001	Severe versus mild/moderate**
Arterial thrombotic event (i.e., leg ischemia)	63 (56)	4 (20)	7 (12)	14 (9)	38 (21)	0.01	Severe versus moderate**
Venous thrombotic event (i.e., deep venous thrombosis)	23 (21)	2 (10)	1 (2)	8 (5)	12 (7)	0.39	n.s
Mechanical thrombotic event	44 (39)	1 (5)	2 (3)	15 (9)	26 (15)	0.07	n.s
28-day mortality	187 (45)	6 (30)	22 (37)	60 (38)	99 (55)	0.002	Severe versus mild*/moderate**

ECMO, extracorporeal membrane oxygenation; RBC, red blood cells

*P < 0.05, **P < 0.01, ***P < 0.001, n.s., non-significant (*P* > 0.05)

^a^Transfusion events, i.e., the total of 226 patients received platelets at 579 days in total

Almost half of the patients (207/419, 49%) suffered a hemorrhagic complication during ECMO. Bleeding patients had a lower platelet count prior ECMO cannulation and during ECMO, when compared to those not bleeding (Additional file 1: Table S5–S6). Among the bleeding patients, 72% received a platelet transfusion during ECMO (*N* = 150); however, in non-bleeding patients, still 36% was transfused (*N* = 76). Majority of these patients (48/76) had a severe thrombocytopenia during ECMO. Bleeding patients, however, received platelets on more occasions and received higher amounts per transfusion event.

### Hemorrhage, thrombocytopenia and platelet transfusion

**Fig. 3:**
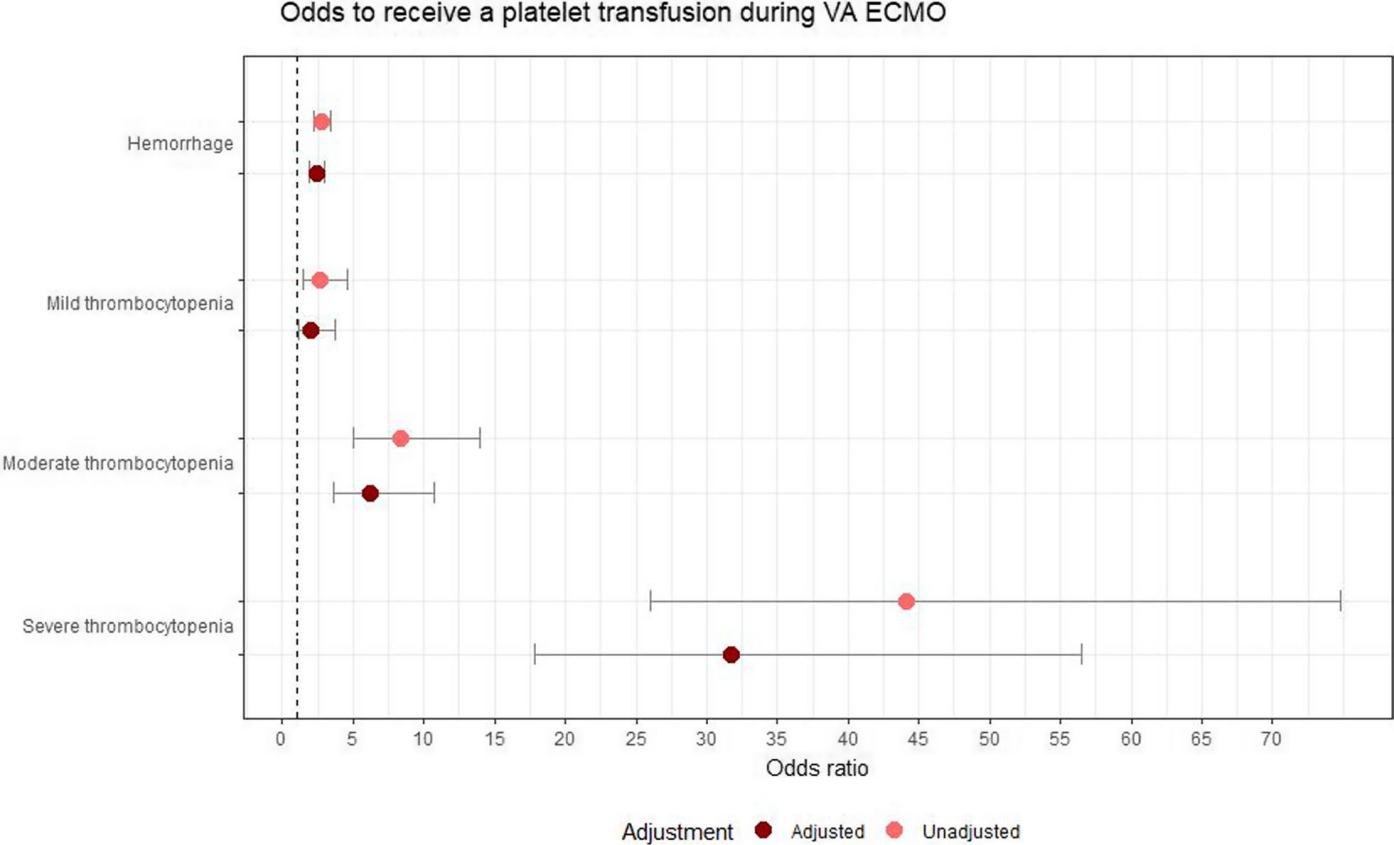
Odds to receive a platelet transfusion during VA ECMO. Odds ratio + 95% confidence interval. In case of adjusted: adjusted for confounding factors. Confounding factors include: sex, age, history of cardiovascular disease, SOFA score at day of ECMO initiation, cannulation site (reference: peripheral), daily aPTT, a thrombotic complication during ECMO and anticoagulation type (reference: unfractionated heparin)

Figure 3 shows the OR to receive a platelet transfusion when either suffering from a hemorrhage or thrombocytopenia, either unadjusted or adjusted for confounding factors. The unadjusted odds (i.e., resulting from the reduced model) to receive a platelet transfusion were highest in the presence of a severe thrombocytopenia (OR 44.1 [95% CI 26–74.8]. For hemorrhage, the unadjusted odds ratio was 2.8 (95% CI 2.3–3.4).

Confounder adjustment decreased the odds for both hemorrhage and all degrees of thrombocytopenia. In the adjusted model, the odds were 31.8 times higher to receive a platelet transfusion in the presence of a severe thrombocytopenia (95% CI 17.9–56.5). The OR for transfusion in the presence of hemorrhage decreased slightly to 2.39 (95% CI 1.9–3.0).

Lastly, the model was performed using an interaction term of hemorrhage and thrombocytopenia (Additional file 1: Table S7). In case a patient suffered from both hemorrhage and thrombocytopenia, the odds to receive a platelet transfusion increased even more up to an OR of 110 (95% CI 34.2–360). In the presence of hemorrhage, the odds for all degrees of severity of thrombocytopenia increased.

## Discussion

Thrombocytopenia, hemorrhage and platelet transfusion are common in patients supported with VA ECMO. This study aimed to describe the occurrence rate and course of thrombocytopenia during VA ECMO, and further assess the association between thrombocytopenia, hemorrhage and platelet transfusion. We report the following clinically relevant findings. First, thrombocytopenia has a high frequency during VA ECMO, whereas almost all patients suffered from a thrombocytopenia. Second, after a global initial decrease in platelet count, stabilization occurred. Third, half of the patients received a platelet transfusion, of which the frequency and number of transfusions administered increased along with the severity of the thrombocytopenia. Severe thrombocytopenia has the strongest association with receiving a platelet transfusion, also after adjustment for confounders. Lastly, the presence of hemorrhage increased the odds to receive a platelet transfusion in all degrees of severity of thrombocytopenia. However, also in the absence of bleeding, a significant part receives a platelet transfusion.

A striking result of this study is that, contrarily to current literature, the incidence of thrombocytopenia in our cohort is significantly higher than previously described. In the systematic review and meta-analysis of Jiritano et al., a pooled incidence was found of 23.2%, based upon the results of six studies [5, 9, 13–17]. However, all these studies were performed in a single-center design, and the heterogeneity among these studies was high. For example, the definition of thrombocytopenia was not always provided [14, 15]. In addition, patient populations consisted of ECPR, refractory cardiogenic shock, bridging for left ventricular assist device and “ECMO in general” [9, 13–17]. This discrepancy in patient populations and methodology could explain part of the differences found with our cohort. Another possible explanation could lie in the fact that the hemorrhage rate reported in our cohort is higher than what is currently stated in large ELSO cohorts, despite using the same definition [1].

We confirm the platelet course consisting of an initial decrease, followed by a stabilization in platelet count, in case a severe thrombocytopenia was not yet present at ECMO initiation [9, 18]. This initial decrease in platelet count can be multi-factorial in etiology, in which VA ECMO adds to the equation by increased platelet consumption in the device itself as well as due to increased shear stress [19–21]. The recovery may be the result of changing patient conditions and the fact that platelets have a mean lifespan of 7 to 10 days [22]. Factors complicating this recovery can include hemorrhage, which is a serious problem that can occur at any moment during ECMO. In addition, previous studies have described an increased incidence of heparin-induced thrombocytopenia (HIT) when compared to similar patient groups [12]. Currently, studies evaluating the degree and type of anticoagulation during ECMO are being performed, adding information in finding the optimal balance among anticoagulation, hemorrhage, and possibly related thrombocytopenia [23].

In our cohort, just over half of the patients received one or more platelet transfusions during VA ECMO, an occurrence rate that is in line with a recent single-center study [8]. Of note, a considerable part of the non-bleeding patients receives a platelet transfusion, of whom a large part at the time of severe thrombocytopenia. In general, indications for platelet transfusion consist of the treatment or prophylaxis of bleeding, such as in case of an invasive procedure or in the presence of severe thrombocytopenia [24–26]. It can be hypothesized that in the transfused non-bleeding patients, the transfusion was administered prophylactically. The benefits of prophylactic platelet transfusion can be questioned. Questions have been raised regarding the increment of platelet transfusion, and whether the benefits and possible risk reduction in bleeding outweigh the downsides of platelet transfusion. Downsides of platelet transfusion include the risk of transfusion-related sequelae in an already vulnerable patient population, next to increasing scarcity and costs of transfusion products [24]. Therefore, a transfusion should be carefully considered. Recently, a randomized controlled trial (RCT) was published focusing on prophylactic platelet transfusion before central venous catheter placement in severely thrombocytopenic critically ill patients in the ICU and hematology ward. They found that withholding platelet transfusion resulted in more placement-related bleeding [27]. As such, a personalized approach may be preferable, differing per patient group and prophylactic indication.

The minimum threshold for platelet transfusion in bleeding and non-bleeding critically ill patients has been a topic of discussion. In ECMO, the variance in the range of thresholds as well as the absolute threshold have been shown to be higher when compared to other critically ill patient populations *not* supported with ECMO [3]. Our study confirms this wide range of thresholds, reflected by the eight different thresholds that were used in our 16 centers. Evidence from randomized trials is scarce, and current guideline recommendations are based on observational studies [25]. Again, which degree of thrombocytopenia to accept, as well as when to transfuse, is a delicate balance. No studies have been performed assessing the threshold for platelet transfusion during VA ECMO, and to our knowledge, none are scheduled in the near future. In addition, during ECMO, there is not only a shortage in absolute platelet count, but impaired platelet function may also be present [28]. Combining both an absolute threshold with platelet function tests may be helpful in the decision when to transfuse.

To our knowledge, this study is the first multicenter collaboration and one of the largest cohort studies focusing on thrombocytopenia during VA ECMO. In addition to the observational data, institutes’ protocols were taken into account, confirming the wide range of thresholds applied in current practice. However, some limitations should be acknowledged. As the data are retrospective, it should be emphasized that no conclusions regarding causality can be drawn. Additionally, no data were collected regarding the indications for platelet transfusion (i.e., prophylactic for a certain procedure), the occurrence of transfusion-related complications such as transfusion-associated circulatory overload or transfusion-related acute lung injury, as well as HIT. Lastly, no data on platelet function or drugs possibly influencing platelet function were collected, as protocols lack standard use of those tests, and differences between centers in the usage and type of function testing were considered too large.

## Conclusions

The occurrence of thrombocytopenia is considerably higher than currently recognized in VA ECMO. Severe thrombocytopenia is an important factor for platelet transfusion, also in the absence of bleeding. It is clear that future research in VA ECMO should focus on the etiology of thrombocytopenia, including the influence of medication during ECMO, as well as evaluating the indications and thresholds for platelet transfusion in bleeding and non-bleeding patients on VA ECMO.

### Supplementary Information

**Additional file 1**. Data definitions events under ECMO. Transfusion questionnaire. Flowchart. **Figure S1**. Platelet course. **Table S1**. Transfusion per center: platelet transfusion and occurrence rates. **Table S2**. Baseline demographics, stratified by transfusion status. **Table S3**. Platelet course, transfusion and complications, stratified by transfusion status. **Table S4**. Transfusion products as stratified per depth of thrombocytopenia. **Table S5**. Baseline demographics, stratified by hemorrhage. **Table S6**. Platelet course, transfusion and complications, stratified by hemorrhage. **Table S7**. Advanced model including interaction term.

## Data Availability

After publication, encrypted data can be requested by contacting the corresponding author. Reasonable data request will be taken in consideration. Additional, related documents can be requested separately.

## Abbreviations

AMC: Academic Medical Center, Amsterdam
aPTT: Activated partial thromboplastin time
CI: Confidence interval
ECMO: Extracorporeal membrane oxygenation
ECPR: Extracorporeal cardiopulmonary resuscitation
ELSO: Extracorporeal Life Support Organization
Hb: Hemoglobin
HIT: Heparin-induced thrombocytopenia
IRB: Institutional Review Board
OR: Odds ratio
SD: Standard deviation
SOFA: Sequential Organ Failure Assessment
VA ECMO: Venoarterial extracorporeal membrane oxygenation
WMO: Wetenschappelijk Medisch Onderzoek (Scientific Medical Research)
