# Supplementary material for: The interaction of thrombocytopenia, hemorrhage, and platelet transfusion in venoarterial extracorporeal membrane oxygenation: a multicenter observational study
Source: Crit Care. 2023 Aug 21;27:321. doi: 10.1186/s13054-023-04612-5 (PMC10441744; doi:10.1186/s13054-023-04612-5)
Supplement: Supplementary file 1 — Additional file 1. Data definitions events under ECMO. Transfusion questionnaire. Flowchart. Figure S1. Platelet course. Table S1. Transfusion per center: platelet transfusion and occurrence rates. Table S2. Baseline demographics, stratified by transfusion status. Table S3. Platelet course, transfusion and complications, stratified by transfusion status. Table S4. Transfusion products as stratified per depth of thrombocytopenia. Table S5. Baseline demographics, stratified by hemorrhage. Table S6. Platelet course, transfusion and complications, stratified by hemorrhage. Table S7. Advanced model including interaction term. [file 13054_2023_4612_MOESM1_ESM.docx]

*Supplementary materials to*

**The interaction between thrombocytopenia, hemorrhage, and platelet transfusion in venoarterial extracorporeal membrane oxygenation: a multicenter observational study**

Senta Jorinde Raasveld, Claudia van den Oord, Jimmy Schenk, Walter M. van den Bergh, Annemieke Oude Lansink-Hartgring, Franciska van der Velde, Jacinta J. Maas, Pablo van de Berg, Roberto Lorusso, Thijs S.R. Delnoij, Dinis Dos Reis Miranda, Erik Scholten, Fabio Silvio Taccone, Dieter F. Dauwe, Erwin De Troy, Greet Hermans, Federico Pappalardo, Evgeny Fominskiy, Višnja Ivancan, Robert Bojčić, Jesse de Metz, Bas van den Bogaard, Dirk W. Donker, Christiaan L. Meuwese, Martin De Bakker, Benjamin Reddi, José P.S. Henriques, Lars Mikael Broman, Dave A. Dongelmans, Alexander P.J. Vlaar.

Table of Contents

[S1. Data definitions events under ECMO 2](#_Toc140217618)

[S2. Transfusion Questionnaire 2](#_Toc140217619)

[S3. Flowchart 5](#_Toc140217620)

[S4. eFigure 1. Platelet course 6](#_Toc140217621)

[S5. eTable 1. Transfusion per center: Platelet transfusion thresholds and occurrence rates 7](#_Toc140217622)

[S6. eTable 2. Baseline demographics, stratified by transfusion status 8](#_Toc140217623)

[S7. eTable 3. Platelet course, transfusion and complications, stratified by transfusion status 9](#_Toc140217624)

[S8. eTable 4. Transfusion products as stratified per depth of thrombocytopenia (NEW) 10](#_Toc140217625)

[S9. eTable 5. Baseline demographics, stratified by hemorrhage 11](#_Toc140217626)

[S10. eTable 6. Platelet course, transfusion and complications, stratified by hemorrhage 12](#_Toc140217627)

[S11. eTable 7. Advanced model including interaction term 13](#_Toc140217628)

# S1. Data definitions events under ECMO

| Acute kidney injury | Increase in serum creatinine by ≥ 26.5 µmol/L within 48 hours; **or** increase in serum creatinine to ≥ 1.5 times baseline, which is known **or** presumed to have occurred within the prior 7 days. |
| --- | --- |
| Thrombotic event (arterial) | Any symptomatic event in the patient (e.g., leg ischemia, stroke) |
| Thrombotic event (mechanical) | Thrombosis in part of ECMO: cannula, pump or oxygenator. |
| Thrombotic event (venous) | Thrombosis in vein(s) (e.g., deep venous thrombosis in upper  or lower extremities). |
| 28-day survival | Alive at day 28 since start ECMO |

# S2. Transfusion Questionnaire

| **Questionnaire: Transfusion & ECMO**  *Center specific protocol and guidelines on anticoagulation and transfusion management during ECMO* |
| --- |

**ECMO** - Extracorporeal membrane oxygenation **aPTT** - Activated partial thromboplastin time [s]

**VA** - Veno-arterial **PT** - Prothrombin time [s]

**VV** - Veno-venous **INR** - International normalized ratio

**Hospital**

1. Name hospital …………………………………………………………………………………………………
2. Country hospital

…………………………………………………………………………………………………

**Transfusion products during ECMO**

*The following questions refer to the standard procedure of transfusion products used during ECMO.*

1. One unit of red blood cells contains ……………………… mL
2. Type of plasma used  Pooled plasma (Omniplasma ®)

Fresh frozen plasma  Other:………………………………………

1. One unit of plasma contains ……………………… mL
2. Type of platelet transfusion used  Apheresis
    (multiple answers possible)  Pooled platelets (5-donor)
     Single buffy coat platelets
     Other:……………………
3. One unit of platelets contains ………………………… mL
4. What coagulation products are commonly used during ECMO?
   (multiple answers possible)  Fibrinogen
     Prothrombin complex concentrate
     Tranexamic acid
     Other:………………

**Transfusion thresholds during VA-ECMO**

1. What unit is used to measure  g/dL  mmol/L  g/L (=mg/mL)
   hemoglobin levels?
2. What is the transfusion threshold for hemoglobin? ………………………………………
3. What unit is used to measure  10^9^/L  mm^3^
   platelet count levels?  Other:………………………………
4. What is the transfusion threshold for platelets? .…………………………………………
5. What is the transfusion threshold for fibrinogen? ...………………………………… gram
6. What is the transfusion threshold for plasma? INR: …………………………………
7. What is the transfusion threshold for vitamin K? INR: …………………………………
8. What is the transfusion threshold for INR: …………………………………
   prothrombin complex concentrate?

**Transfusion thresholds during VV-ECMO**

1. What is the transfusion threshold for hemoglobin? ………………………………………
2. What is the transfusion threshold for platelets? ……………..……………………………
3. What is the transfusion threshold for fibrinogen? …………………………………………
4. What is the transfusion threshold for plasma? INR: …………………………………
5. What is the transfusion threshold for vitamin K? INR: …………………………………
6. What is the transfusion threshold for INR: …………………………………
   prothrombin complex concentrate?

**Anticoagulation during VA-ECMO**

1. What type of anticoagulation is used during VA-ECMO?
    Unfractionated heparin

Low molecular weight heparin
 Direct thrombin inhibitors (e.g. bivalirudin, argatroban)
 Antiplatelet drugs
 Other:…………………………………………………………………………………

1. What tests are used to monitor anticoagulation?
    aPTT

anti-Xa
 INR or PT
 Other: …………………………………………………………………………………

1. What thresholds are used to monitor anticoagulation therapy?
   Minimum: ………………………………………………………………………………………
   Maximum: ……………………………………………………………………………………..

**Anticoagulation during VV-ECMO**

1. What type of anticoagulation is used during VV-ECMO?
    Unfractionated heparin

Low molecular weight heparin
 Direct thrombin inhibitors (e.g. bivalirudin, argatroban)
 Antiplatelet drugs
 Other: ………………………………………………………………………………

1. What tests are used to monitor anticoagulation?
    aPTT

anti-Xa
 INR or PT
 Other: ………………………………………………………………………………

1. What are the thresholds for monitoring anticoagulation?
   Minimum: …………………………………………………………………………………..
   Maximum: ………………………………………………………………………………….

S3. Flowchart


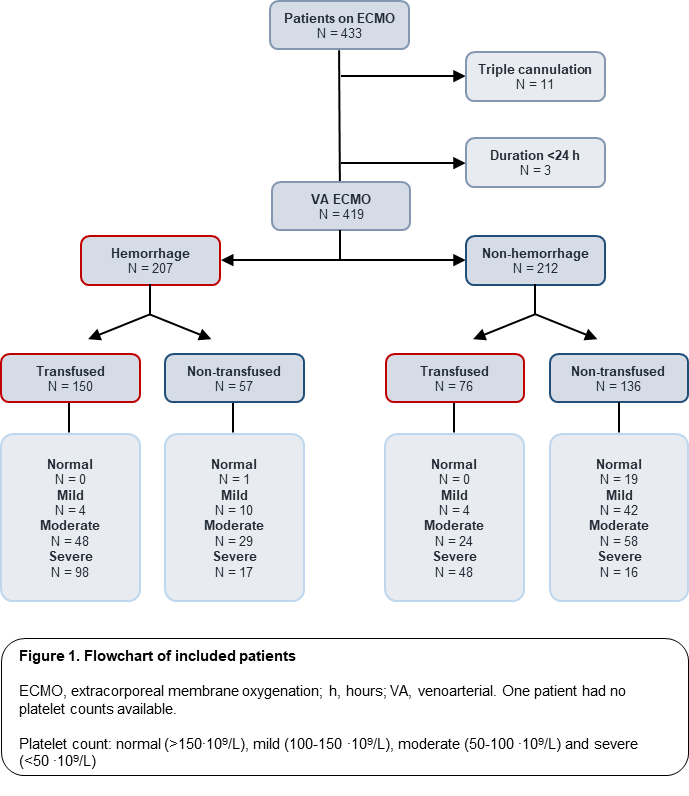


# S4. eFigure 1. Platelet course


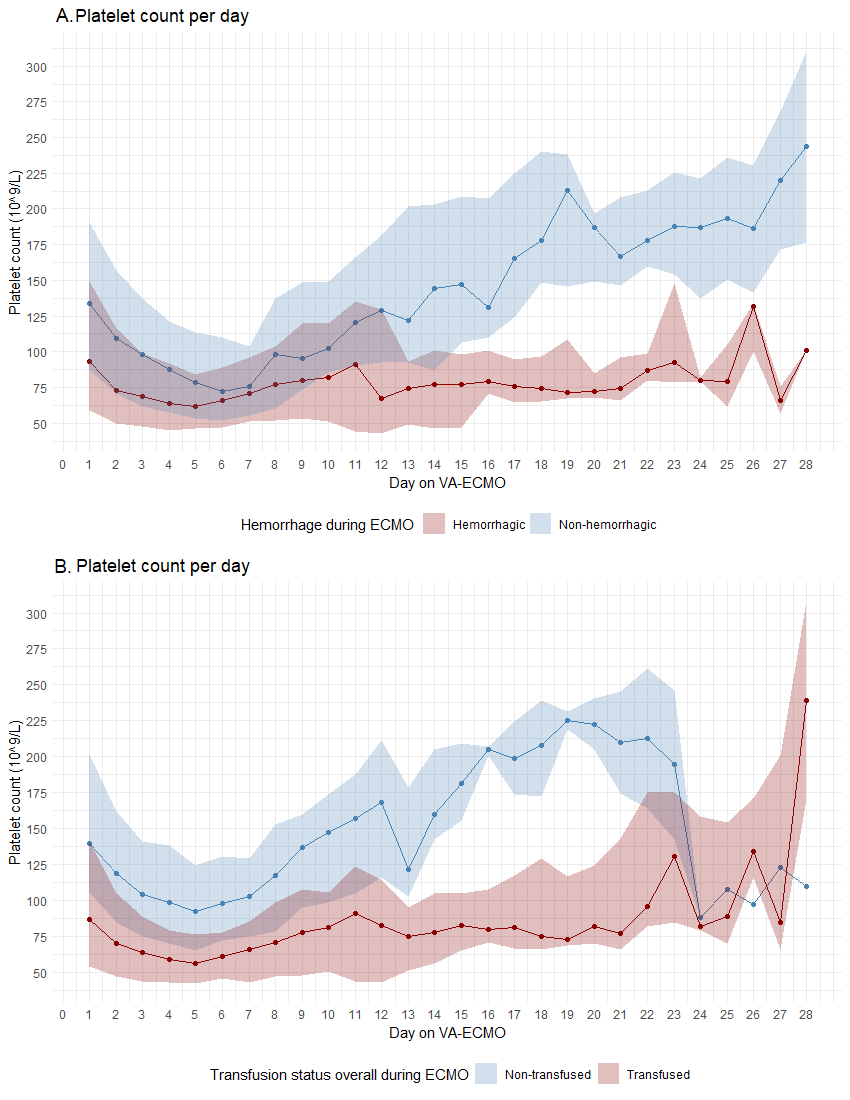


**Panel A** describes the platelet count as median and interquartile range when stratified by whether the patient suffered a hemorrhage during VA-ECMO. **Panel B** describes the platelet count as median and interquartile range when stratified by whether the patient received one or more platelet transfusions during VA-ECMO.

# S5. eTable 1. Transfusion per center: Platelet transfusion thresholds and occurrence rates

| **eTable 1. Transfusion per center: Platelet transfusion** | | | | | | |
| --- | --- | --- | --- | --- | --- | --- |
| Center | Anticoagulation | Platelet threshold | Platelet threshold: comments | % transfused | % hemorrhage | mean nadir platelet count |
| 1 | Bivalirudin (aPTT 50 - 65 sec) | 30* | * bleeding | 25% | 41% | 49 |
| 2 | Unfractionated heparin (aPTT 1.5 - 2.0 ULN) | 20 - 50 | - | 50% | 39% | 73 |
| 3 | Unfractionated heparin (aPTT 1.5 - 1.8 ULN) | 50 | - | 56% | 44% | 60 |
| 4 | Unfractionated heparin (aPTT 50 - 80 sec) | 50 | - | 45% | 40% | 63 |
| 5 | Unfractionated heparin (aPTT 50 - 70 sec) | 50 | - | 50% | 60% | 68 |
| 6 | Unfractionated heparin (aPTT 50 - 80 sec) | 50 | - | 48% | 48% | 75 |
| 7 | Unfractionated heparin (aPTT 40 - 50 sec) | 50 / 100* | * bleeding | 56% | 59% | 75 |
| 8 | Unfractionated heparin (aPTT 40 - 80 sec) | 50-100 | based on clinical scenario | 71% | 61% | 64 |
| 9 | Unfractionated heparin (aPTT 60 - 80 sec) | 50 | - | 67% | 33% | 57 |
| 10 | Unfractionated heparin (aPTT 1.8 - 2.5 ULN) | 50 | - | 60% | 48% | 61 |
| 11 | Unfractionated heparin (aPTT 50 - 70 sec) | 50 / 80* | * bleeding | 58% | 55% | 75 |
| 12 | Unfractionated heparin (aPTT 50 - 55 sec) | 60 | - | 57% | 57% | 83 |
| 13 | Unfractionated heparin (aPTT 60 - 80 sec) | 80 | - | 33% | 67% | 126 |
| 14 | Unfractionated heparin (ACT 160 - 220 sec) | 80-100 | - | 92% | 58% | 56 |
| 15 | Unfractionated heparin (1.7 - 2.0 ULN) | 100* | * bleeding, no threshold available in non-bleeding | 71% | 24% | 89 |
| 16 | Unfractionated heparin (aPTT 60 - 90 sec) | 10 / 50* | * bleeding | 81% | 54% | 54 |

# S6. eTable 2. Baseline demographics, stratified by transfusion status

| **eTable 2. Baseline demographics as stratified by transfusion-status** | | | | |
| --- | --- | --- | --- | --- |
|  | **Overall (N = 419)** | **Transfused (N = 226)** | **Non-transfused (N = 193)** | **P-value** |
| Age, years | 57 [47-66] | 59 [50-68] | 54 [44-64] | <0.001 |
| BMI, kg/m^2^ | 27.0 [24.3-30.6] | 27.2 [24.2-30.4] | 26.7 [24.5-30.7] | 0.73 |
| Female | 154 (37) | 100 (44) | 54 (28) | 0.001 |
| **Medical history** |  |  |  |  |
| Hypertension | 139 (62) | 84 (27) | 55 (28) | 0.97 |
| Diabetes mellitus | 68 (30) | 37 (17) | 31 (16) | 0.25 |
| Myocardial infarction | 76 (34) | 48 (21) | 28 (15) | 0.72 |
| Asthma/COPD | 37 (55) | 16 (7) | 21 (11) | 0.052 |
| Chronic kidney disease | 25 (6) | 18 (8) | 7 (4) | 0.10 |
| **Day of ECMO initiation** |  |  |  |  |
| SOFA-score | 11 [8-13] | 12 [9-14] | 11 [8-13] | 0.14 |
| Lactate, mmol/L | 5.2 [2.4-10.0] | 5.6 [2.6-9.5] | 5.2 [2.2-10.2] | 0.88 |
| Hb, g/dL | 11.6 [9.5-13.5] | 10.7 [9.2-13.2] | 12.0 [10.2-14.1] | <0.01 |
| Platelet count | 179 [119-253] | 157 [100-212] | 221 [155-305] | <0.001 |
| Different in platelet count day of initiation - first day of ECMO nadir platelet count | 49 [6-102] | 51 [17-110] | 40 [0-93] | 0.35 |
| **ECMO characteristics** |  |  |  |  |
| Duration, days | 5 [3 - 8] | 6 [4-9] | 4 [3-6] | <0.001 |
| Second run | 38 (9) | 27 (12) | 11 (6) | 0.04 |
| Peripheral cannulation configuration | 356 (86) | 174 (78) | 182 (94) | <0.001 |
| Surgical cannulation | 227 (56) | 146 (66) | 81 (43) | <0.001 |
| Distal leg perfusion cannula | 288 (71) | 145 (66) | 143 (76) | 0.05 |
| ECPR | 107 (26) | 38 (17) | 69 (36) | <0.001 |
| Main reason of ECMO initiation |  |  |  | <0.001 |
| Acute myocardial infarction | 117 (28) | 57 (25) | 60 (31) |  |
| Post-cardiotomy | 113 (27) | 83 (37) | 106 (55) |  |
| Other | 189 (45) | 86 (38) | 27 (14) |  |
| **Abbreviations:** BMI, body mass index; COPD, chronic obstructive pulmonary disease; ECMO, extracorporeal membrane oxygenation; ECPR, extracorporeal cardiopulmonary resuscitation; Hb, hemoglobin; SOFA, sequential organ failure assessment * P <0.05, **, P<0.01, *** P<0.001 | | | | |

# S7. eTable 3. Platelet course, transfusion and complications, stratified by transfusion status

| **eTable 3. Platelet course, transfusion and complications, stratified by transfusion status** | | | | |
| --- | --- | --- | --- | --- |
|  | **Overall  (N = 419)** | **Transfused  (N = 226)** | **Non-transfused  (N = 193)** | **P-value** |
| **Laboratory values** |  |  |  |  |
| Platelet count prior | 179 [119-253] | 157 [100-212] | 221 [155-305] | <0.001 |
| Difference platelet count before-after cannulation | 49 [6-102] | 51 [17-110] | 40 [0-93] | 0.350 |
| During ECMO: minimal platelet count | 56 [37-89] | 42 [28-57] | 89 [58-116] | <0.001 |
| During ECMO: mean platelet count | 89 [63-129] | 73 [57-93] | 119 [85-149] | <0.001 |
| Day of nadir platelet count |  |  |  |  |
| Nadir platelet count, categorical |  |  |  | <0.001 |
| Normal (>150) | 20 (5) | 0 (0) | 20 (10) |  |
| Mild (100 - 150) | 60 (14) | 8 (4) | 52 (27) |  |
| Moderate (50 - 100) | 159 (38) | 72 (32) | 87 (45) |  |
| Severe (<50) | 179 (43) | 146 (65) | 33 (17) |  |
| **Platelet transfusion** |  |  |  |  |
| Proportion transfused | 226 (54) | 226 (100) | 0 (0) | - |
| Total platelets transfused | 4 [2-7] | 4 [2-7] | - | - |
| No. of days receiving platelet transfusion | 2 [1-3] | 2 [1-3] | - | - |
| Number of platelets transfused per day on ECMO (units) | 0.63 [0.33-1.14] | 0.63 [0.33-1.14] | - | - |
| Concomitant RBC transfusion among the transfused | 221 (98) | 221 (98) | - | - |
| **Complications** |  |  |  |  |
| Acute kidney injury | 242 (58) | 143 (63) | 99 (52) | 0.020 |
| Hemorrhage | 207 (49) | 150 (66) | 57 (30) | <0.001 |
| Thrombotic event | 112 (27) | 74 (33) | 38 (20) | 0.004 |
| Arterial thrombotic event (i.e., leg ischemia) | 63 (56) | 40 (18) | 23 (12) | 0.130 |
| Venous thrombotic event (i.e., deep venous thrombosis) | 23 (21) | 15 (7) | 8 (4) | 0.367 |
| Mechanical thrombotic event | 44 (39) | 31 (14) | 13 (7) | 0.031 |
| 28-day mortality | 188 (45) | 113 (50) | 75 (39) | 0.03 |
| ^a^ Transfusion events, i.e., the total of 226 patients received platelets at 579 days in total. * P <0.05, **, P<0.01, *** P<0.001 **Abbreviations:** ECMO, extracorporeal membrane oxygenation; RBC, red blood cells | | | | |

# S8. eTable 4. Transfusion products as stratified per depth of thrombocytopenia (NEW)

| **eTable 4. Transfusion of other blood and procoagulant products** | | | | | |
| --- | --- | --- | --- | --- | --- |
|  | **Normal (>150) (N = 20)** | **Mild (100-150) (N = 60)** | **Moderate  (50-100) (N = 159)** | **Severe (<50) (N = 179)** | **P-value** |
| **Red blood cell transfusion**  Proportion transfused  Total units transfused during ECMO run | 9 (45)  3 (2 – 4) | 45 (75)  3 (2 – 7) | 145 (91)  9 (5-14) | 174 (97)  15 (8 – 24) | <0.001 <0.001 |
| **Plasma transfusion**  Proportion transfused  Total units transfused during ECMO run | 3 (15)  1 (1 – 2) | 18 (30)  2 (2 – 4) | 91 (57)  7 (4-11) | 134 (75)  8 (3 – 15) | <0.001 <0.001 |
| **Prothrombin complex concentrate administration**  Proportion received  Total IE received during ECMO run | 1 (5)  1500 (1500 – 1500) | 4 (7)  750 (438 – 1500) | 4 (3)  2750 (500 – 6375) | 19 (11)  1500 (750 – 2500) | 0.03  0.70 |
| **Fibrinogen suppletion**  Proportion received  Total grams received during ECMO run | 1 (5) 4 (4 – 4) | 3 (5)  2 (1.5 – 3) | 28 (18)  2 (2 – 4.4) | 50 (28)  3 (2 – 5) | <0.001  0.46 |
| **Tranexamic acid administration**  Proportion received  Total mg received during ECMO run | 0 (0)  0 (0) | 3 (5)  2000 (2000 – 2500) | 29 (18)  2000 (1000 – 2000) | 38 (21)  2000 (2000 – 4000) | <0.01  <0.01 |

Proportion of the population receiving either red blood cells, plasma, prothrombin complex concentrate, fibrinogen or tranexamic acid during VA-ECMO, stratified per severity of thrombocytopenia. Please note that the denominator in this table differs from table 2. In table 2, it is the proportion of the patients that also received a platelet transfusion; in this table the denominator is the thrombocytopenic subgroup, independent of receiving a platelet transfusion or not. The total amount received is calculated for the patients that have received that certain blood or procoagulant product. Unadjusted P-value provided.

# S9. eTable 5. Baseline demographics, stratified by hemorrhage

| **eTable 5. Baseline demographics as stratified by bleeding-status** | | | | |
| --- | --- | --- | --- | --- |
|  | **Overall  (N = 419)** | **Bleeding (N = 207)** | **Non-bleeding (N = 212)** | **P-value** |
| Age, years | 57 [47-66] | 60 [50-68] | 56 [45-64] | <0.01 |
| BMI, kg/m^2^ | 27.0 [24.3-30.6] | 26.9 [24.4-30.1] | 27.1 [24.2-30.9] | 0.88 |
| Female | 154 (37) | 89 (43) | 65 (31) | 0.01 |
| **Medical history** |  |  |  |  |
| Hypertension | 139 (62) | 77 (37) | 62 (29) | 0.76 |
| Diabetes mellitus | 68 (30) | 36 (17) | 32 (15) | 0.91 |
| Myocardial infarction | 76 (34) | 43 (22) | 33 (16) | 0.72 |
| Asthma/COPD | 37 (55) | 16 (8) | 21 (10) | 0.56 |
| Chronic kidney disease | 25 (6) | 19 (9.2) | 6 (3) | 0.01 |
| **Day of ECMO initiation** |  |  |  |  |
| SOFA-score | 11 [8-13] | 11 [9-14] | 11 [8-13] | 0.07 |
| Lactate, mmol/L | 5.2 [2.4-10.0] | 5.9 [2.5-11] | 4.45 [2.3-9.0] | 0.31 |
| Hb, g/dL | 11.6 [9.5-13.5] | 11.2 [9.3-13.4] | 11.8 [9.8-13.9] | 0.13 |
| Platelet count | 179 [119-253] | 161 [101-224] | 207 [139-294] | <0.001 |
| Different in platelet count day of initiation - first day of ECMO nadir platelet count | 49 [6-102] | 50 [11-103] | 42 [3-100] | 0.91 |
| **ECMO characteristics** |  |  |  |  |
| Duration, days | 5 [3 - 8] | 6 [4-9] | 4 [3-7] | <0.001 |
| Second run | 38 (9) | 27 (13) | 11 (5) | <0.01 |
| Peripheral cannulation configuration | 356 (86) | 165 (81) | 191 (90) | 0.01 |
| Surgical cannulation | 227 (56) | 127 (63) | 100 (48) | <0.01 |
| Distal leg perfusion cannula | 288 (71) | 135 (67) | 153 (74) | 0.17 |
| ECPR | 107 (26) | 55 (27) | 52 (25) | 0.60 |
| Main reason of ECMO initiation |  |  |  | 0.02 |
| Acute myocardial infarction | 117 (28) | 57 (28) | 60 (28) |  |
| Post-cardiotomy | 113 (27) | 82 (40) | 107 (51) |  |
| Other | 189 (45) | 68 (33) | 45 (21) |  |
| **Abbreviations:** BMI, body mass index; COPD, chronic obstructive pulmonary disease; ECMO, extracorporeal membrane oxygenation; ECPR, extracorporeal cardiopulmonary resuscitation; Hb, hemoglobin; SOFA, sequential organ failure assessment * P <0.05, ** P<0.01, *** P<0.001 | | | | |

# S10. eTable 6. Platelet course, transfusion and complications, stratified by hemorrhage

| **eTable 6. Platelet course, transfusion and complications, stratified by hemorrhage** | | | | |
| --- | --- | --- | --- | --- |
|  | **Overall  (N = 419)** | **Bleeding  (N = 207)** | **Non-bleeding  (N = 212)** | **P-value** |
| **Laboratory values** |  |  |  |  |
| Platelet count prior | 179 [119-253] | 161 [101-224] | 207 [139-294] | <0.001 |
| Difference platelet count before-after cannulation | 49 [6-102] | 50 [11-103] | 42 [2.5-100] | 0.92 |
| During ECMO: minimal platelet count | 56 [37-89] | 46 [29-67] | 72 [45-110] | <0.001 |
| During ECMO: mean platelet count | 89 [63-129] | 77 [59-105] | 105 [74-141] | <0.001 |
| Nadir platelet count, categorical |  |  |  | <0.001 |
| Normal (>150) | 20 (5) | 1 (<1) | 19 (9) |  |
| Mild (100 - 150) | 60 (14) | 14 (7) | 46 (22) |  |
| Moderate (50 - 100) | 159 (38) | 77 (37) | 82 (39) |  |
| Severe (<50) | 179 (43) | 115 (56) | 64 (30) |  |
| **Platelet transfusion** |  |  |  |  |
| Proportion transfused | 226 (54) | 150 (72) | 76 (36) | <0.001 |
| Total platelets transfused | 4 [2-7] | 4 [2-8] | 2 [1-4] | <0.001 |
| No. of days receiving platelet transfusion | 2 [1-3] | 2 [1-3] | 1 [1-2] | <0.001 |
| Number of platelets transfused per day on ECMO (units) | 0.63 [0.33-1.14] | 0.72 [0.40-1.25] | 0.50 [0.25-1] | <0.01 |
| Number of platelets transfused per transfusion day (units)* |  |  |  | <0.001 |
| 1 | 306/579 (53) | 217/434 (50) | 89/145 (61) |  |
| 2-3 | 179/579 (31) | 147/434 (34) | 32/145 (22) |  |
| ≥4 | 94/579 (16) | 70/434 (16) | 24/145 (17) |  |
| Concomitant RBC transfusion among the transfused | 221 (98) | 149 (99) | 72 (95) | 0.08 |
| **Complications** |  |  |  |  |
| Acute kidney injury | 242 (58) | 125 (60) | 117 (56) | 0.36 |
| Hemorrhage | 207 (49) | 207 (100) | 0 (0) | - |
| Thrombotic event | 112 (27) | 63 (30) | 49 (23) | 0.11 |
| Arterial thrombotic event (i.e., leg ischemia) | 63 (56) | 40 (19) | 23 (11) | 0.02 |
| Venous thrombotic event (i.e., deep venous thrombosis) | 23 (21) | 12 (6) | 11 (5) | 0.95 |
| Mechanical thrombotic event | 44 (39) | 22 (11) | 22 (10) | 1.00 |
| 28-day mortality | 188 (45) | 110 (53) | 78 (37) | 0.001 |
| ^a^ Transfusion events, i.e., the total of 226 patients received platelets at 579 days in total. * P <0.05, ** P<0.01, *** P<0.001 **Abbreviations:** ECMO, extracorporeal membrane oxygenation; RBC, red blood cells | | | | |

# S11. eTable 7. Advanced model including interaction term

|  | **Odds ratio** | **Lower limit (95% CI)** | **Upper limit (95% CI)** |
| --- | --- | --- | --- |
| **Hemorrhage, no thrombocytopenia** | 5.7 | 1.6 | 20.2 |
| **Hemorrhage, thrombocytopenia:**   - **Mild** - **Moderate** - **Severe** | - 10.2 - 24.3 - 110 | - 3.1 - 7.6 - 34.2 | - 33.7 - 77.8 - 360 |
| **NO hemorrhage, thrombocytopenia:**   - **Mild** - **Moderate** - **Severe** | - 3.0 - 10.9 - 75.1 | - 0.8 - 3.3 - 22.6 | - 10.8 - 35.6 - 250 |
